# Synchronous Pulmonary Neoplasms: A Chance Occurrence or is There More Than Meets the Eye?

**DOI:** 10.7759/cureus.2162

**Published:** 2018-02-06

**Authors:** Uroosa Ibrahim, Amina Saqib, Manisha Pant, Gwenalyn Garcia, Marcel Odaimi

**Affiliations:** 1 Department of Hematology and Oncology, Staten Island University Hospital; 2 Pulmonary/Critical Care, Staten Island University Hospital; 3 Medicine, Staten Island University Hospital

**Keywords:** synchronous neoplasm, squamous cell lung cancer, balt lymphoma, non-hodgkin lymphoma, nsclc

## Abstract

Primary bronchus-associated lymphoid tissue (BALT) lymphoma comprises 5% of non-Hodgkin’s lymphoma (NHL) and usually has an indolent course. Synchronous primary lung cancers with BALT lymphoma are seldom seen in patients with adenocarcinoma of the lung. Synchronous squamous cell carcinoma (SCC) and BALT lymphoma is an extremely rare occurrence. We report an unusual case of stage 4 BALT lymphoma requiring treatment that revealed an underlying ipsilateral mass causing a diagnostic dilemma. An 84-year-old female with a history of systemic lupus erythematosus, deep vein thrombosis, and thrombotic microangiopathy presented to the hospital with cough and dyspnea on exertion. A chest X-ray revealed right hemi-thorax opacification and computed tomography (CT) of the chest showed a large right effusion and a soft tissue density extending into the proximal right bronchus. She required repeated thoracentesis until the pleural fluid analysis showed the presence of small lymphocytes and bronchial washings revealed an abnormal B cell population consistent with extranodal marginal zone lymphoma. The patient received four cycles of bendamustine and rituximab resulting in near-complete resolution of the effusion. Four months from diagnosis, imaging showed an increase in the size of the soft tissue density with pathologic fluorodeoxyglucose (FDG) uptake on positron emission tomography (PET). A CT-guided biopsy was consistent with squamous cell lung cancer (SCLC) and radiotherapy was started for clinical stage 2 disease since the patient was not a surgical candidate. BALT lymphoma is a low-grade malignancy classified as extranodal marginal zone lymphoma with a five-year survival rate of over 80%. Several cases of synchronous lung adenocarcinoma and BALT lymphoma have been described. However, our case is among the rare few cases of synchronous occurrence of SCLC with BALT lymphoma. This report highlights the challenges associated with establishing an accurate and timely diagnosis.

## Introduction

Primary bronchus-associated lymphoid tissue (BALT) lymphoma is a distinct type of mucosa-associated lymphoid tissue (MALT) lymphoma that comprises of B cells and is recognized as extranodal marginal zone B cell lymphoma of MALT type in the World Health Organization classification. It comprises about 5% of all non-Hodgkin’s lymphoma (NHL) [1]. Among lung cancers, squamous cell carcinoma (SCC) is the second most common histologic subtype of non-small cell lung carcinoma (NSCLC). The co-existence of BALT lymphoma and SCC in the same anatomical site is very rare and can lead to a missed diagnosis if the possibility of co-occurrence is not entertained. We report a unique case of synchronous occurrence of BALT lymphoma and SCC causing a diagnostic dilemma.

## Case presentation

**Figure 1:**
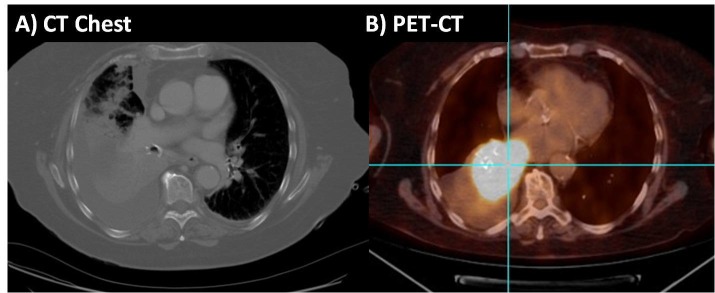
A) Chest computed tomography (CT) scan demonstrating right lower lobe consolidation with moderate to large sized right pleural effusion and B) positron emission tomography-computed tomography (PET-CT) scan demonstrating increased fluorodeoxyglucose (FDG) uptake in the heterogeneous right lower lobe pulmonary mass involving bronchus intermedius and right lower lobe bronchus

We present the case of an 84-year-old female who presented with cough productive of clear phlegm, dyspnea on exertion, and right-sided chest pain. Her past medical history included systemic lupus erythematosus, thrombotic microangiopathy, deep venous thrombosis, coronary artery disease, hypertension, and hypercholesterolemia. She was a former smoker with a 60 pack-year smoking history. Family history was noncontributory. A physical examination revealed decreased breath sounds over the right lower lung field with associated scattered rales. A chest X-ray revealed right lower lobe opacity with right pleural effusion. A computed tomography (CT) of the chest showed right lower lobe consolidation with moderate to large sized right pleural effusion and associated right hilar and mediastinal lymphadenopathy, thought to be reactive in nature. Diagnostic and therapeutic thoracentesis was performed, and she was empirically started on antibiotics for possible pneumonia. The pleural fluid analysis was consistent with a transudate with no malignant cells on cytology. She was subsequently discharged to follow up as an outpatient. After a month, she was readmitted for worsening dyspnea, cough, and hemoptysis. Chest X-ray showed increased right-sided pleural effusion and CT chest revealed large right pleural effusion with a new appearing soft tissue density filling the bronchus with associated atelectasis (Figure 1). The patient underwent another diagnostic and therapeutic thoracentesis in addition to bronchoscopy and bronchioalveolar lavage. A biopsy could not be performed because she was unable to tolerate the entire procedure.

Pleural fluid cytology showed an abnormal B cell population consistent with a B cell lymphoproliferative disorder. Of the 93% total viable cells, approximately 51% were T cells and 42% were B cells. Virtually all of the B cells expressed CD19, CD20, PAX5, CD43 and CD38 dim. Kappa light chain was equivocal (dim/negative). The cells were negative for CD5, CD10, CD11C, CD22, CD23, BCL6, cyclin D1, and surface lambda light chain. No abnormal T-cell population was identified. Pathology from bronchial brushings also revealed a monoclonal kappa B cell population lacking CD5 or CD10 expression.

With the presumed diagnosis of BALT lymphoma, the patient was started on bendamustine and rituximab. After four cycles of chemotherapy, a positron emission tomography/computed tomography (PET-CT) scan demonstrated increased fluorodeoxyglucose (FDG) uptake in the heterogenous right lower lobe pulmonary mass involving bronchus intermedius and right lower lobe bronchus (Figure 1). Various possibilities were considered at this point including the progression of marginal zone lymphoma or a second primary lung cancer. Pathology from a subsequent CT-guided core biopsy revealed squamous cell lung cancer (SCLC), consistent with clinical stage 2A disease. Given the advanced age of the patient and comorbidities, she was not a surgical candidate. Radiotherapy was pursued and she received a total of 5000 cGy in 20 fractions with symptomatic improvement following which she developed a spontaneous pneumothorax deemed to be secondary to radiotherapy. It was managed by suctioning with pleurX vacutainer. Two months later, she developed loose watery diarrhea, increased lethargy, and a decline in overall functional status. Given the debility, multiple hospitalizations, and overall poor prognosis, a decision for hospice care was made.

## Discussion

Synchronous existence of primary lung cancer with BALT lymphoma is relatively uncommon. The estimated frequency of synchronous lung cancers has been reported to be from 0.2% to 8% [2]. Synchronous multiple primary cancer is defined as two or more tumors occurring within six months of each other and include the following diagnostic criteria: a) the cancer must be clearly malignant as determined by histologic evaluation; b) each cancer must be geographically separate and distinct; and c) the possibility that the second tumor represents a metastasis should be excluded.

SCLC represents about 25%–30% of all lung cancers most of which present as central tumors [3]. After adenocarcinoma, SCLC is the most frequent histologic subtype in NSCLC. It arises from bronchial epithelial cells through squamous metaplasia/dysplasia and is characterized by keratinization and/or intercellular bridges [4]. Risk factors for SCLC include cigarette smoking, second-hand smoke, ionizing radiation, air pollution, occupational exposure to asbestos, silica, and arsenic [5].

BALT lymphoma is a low-grade primary B cell lymphoma, a distinct subtype of NHL. It is the most common type of primary pulmonary lymphoma and develops from mucosa-associated lymphoid tissue [6]. BALT lymphoma is associated with various connective tissue diseases including rheumatoid arthritis, systemic lupus erythematosus (SLE), and especially, Sjögren's syndrome. Other associated factors include infectious causes such as mycobacterium avium complex, mycobacterium tuberculosis, Epstein-Barr virus, and human herpesvirus 8 (HHV-8) [7]. In our case, the patient’s history of systemic lupus erythematosus may be a relevant association. 

Pathological diagnosis is required for both the hematologic and solid tumor malignancy and may be obtained by bronchial or transbronchial biopsies, CT-guided percutaneous needle biopsies or surgical biopsies [8]. In our case, we obtained bronchial and pleural fluid samples, which revealed IgH gene arrangement demonstrating monoclonality of the B cell population. SCLC was diagnosed by a CT-guided needle biopsy.

As described in our case, the lymphomatous pleural effusion initially masked the concurrent tumor and the possibility that the soft tissue density represented another tumor was not entertained following the diagnosis of BALT lymphoma until the repeat PET-CT scan during clinical follow up demonstrated an increased pathologic FDG uptake consistent with biologic tumor activity. This emphasizes the fact that tissue biopsy must be taken separately if there are concurrent lesions and the possible existence of another cancer should be considered if the clinical course aggravates despite treatment.

Synchronous malignancies in the same anatomical site are very unusual [9-10]. This case is one of those scarce reports of the simultaneous occurrence of BALT lymphoma and SCLC in the same anatomical site. However, the cause of synchronous lesions in our case is uncertain. Possible causes may include shared risk factors and genetic/chromosomal alterations contributing to the development of both tumors. Although the cause in the presented case in unknown, we can speculate that certain risk factors like smoking for SCLC and history of autoimmune disease for BALT lymphoma could lead to the existence of both tumors in our case. Reporting of cases of synchronous lung cancers is necessary to elucidate other associated factors.

## Conclusions

This report highlights the challenges associated with establishing an accurate diagnosis for these rare neoplasms. Although there are reports of synchronous occurrence of solid tumors with hematological malignancies, no pathological correlation has been established. This raises the question of whether this synchrony is just a chance occurrence or is there an underlying shared pathogenesis.
